# Readiness for hospital discharge and influencing factors: a cross-sectional study on patients discharged with tubes from the department of hepatobiliary surgery

**DOI:** 10.1186/s12893-021-01119-0

**Published:** 2021-03-08

**Authors:** Jingyu Qian, Miaofang Qian, Yanyan Ren, Linyan Ye, Fangfang Qian, Linlin Jin, Lili Chen, Haixia Xu

**Affiliations:** 1 Department of Hepatobiliary Surgery, Shaoxing Second Hospital, Zhejiang, Shaoxing, China; 2 Department of Operation Rooms, Shaoxing People’s Hospital, Shaoxing People’s Hospital (Shaoxing Hospital, Zhejiang University School of Medicine), Zhejiang, Shaoxing, China; 3 Department of General Surgery, Shaoxing People’s Hospital, Shaoxing People’s Hospital (Shaoxing Hospital, Zhejiang University School of Medicine), 568 Zhongxing North Road, Shaoxing, 312000 Zhejiang China

**Keywords:** Department of hepatobiliary surgery, Discharged patients with tubes, Readiness for hospital discharge, Influencing factors

## Abstract

**Background:**

To investigate the readiness for hospital discharge of patients discharged with tubes from the department of hepatobiliary surgery and to explore the influencing factors.

**Methods:**

A cross-sectional survey was conducted for the 161 patients with tubes who were discharged from the department of hepatobiliary surgery of Shaoxing Second Hospital by using the modified Chinese version of Readiness for Hospital Discharge Scale (RHDS) and Quality of Discharge Teaching Scale (QDTS). General data of the patients, such as gender, age, BMI (body mass index), and educational level, were collected.

**Results:**

According to the statistical results, the total score of the RHDS was 142.40 ± 23.98, and that of the QDTS was 148.14 ± 17.74. Multiple linear step-wise regression analysis revealed that the total score of the QDTS, residence and educational level were the independent influencing factors of the readiness for hospital discharge (*p* < 0.05).

**Conclusion:**

The level of the readiness for hospital discharge of the 161 discharged patients with tubes from the department of hepatobiliary surgery was in the middle and lower level. For the patients who are far away from the hospital and have a low education level, we should pay more attention to health education and discharge teaching, so as to improve the readiness for hospital discharge of relatively vulnerable patients, reduce the incidence of adverse events after discharge with tubes, and ensure the health and safety of patients.

## Background

With the wide implementation of fast track surgery in clinical practice, the improvement of tiered medical services, and the increasingly strict assessment of the average length of stay of patients by medical institutions at all levels [1–6], many patients treated in the department of hepatobiliary surgery often leave hospital with tubes [7–11]. These tubes mainly include T-tubes [12], percutaneous transhepatic cholangial drainage (PTCD) tubes, and percutaneous transhepatic gallbladder drainage (PTGD) tubes, and some of the patients need to return to the hospital for extubation several days after discharge or go to the lower level hospital and stay at home for continuing rehabilitation treatment. The shortening of the average length of hospital stay will inevitably lead to many patients' poor mastery of the relevant knowledge of tube maintenance and complications observation when they are discharged, which may lead to the occurrence of adverse events such as accidental extubation, tube-related infection, tube blockage, etc. and thus affect the prognosis and even aggravate the disease. Therefore, it is very important to evaluate the readiness for hospital discharge of discharged patients with tubes. The concept of readiness for hospital discharge [13] was put forward by a British scholar called Fenwick in 1979 [14]. It is used to comprehensively evaluate the health status of patients, including the state of physical and psychological readiness or the process of gradual preparation over time. Its connotation is also constantly improved in the course of development. According to Weiss and other colleagues [15], readiness for hospital discharge refers to the ability of patients or (and) their families to leave the hospital, return to family and society, and further recover, after the physiological, psychological and social conditions of the patients were comprehensively evaluated by medical staff. A Readiness for Hospital Discharge Scale (RHDS) was thereby invented which was more commonly used in western countries. After that, Lin [16], a Taiwan scholar, and Zhao [17], a doctor working in the West China Hospital, developed a modified Chinese version of the RHDS considering local conditions, and conducted validity test and reliability measurement. This scale provided theoretical support, scale template and data model for the follow-up readiness evaluation in various patients. The purpose of this study is to explore the status quo of the readiness for hospital discharge of the patients discharged with tubes from the department of hepatobiliary surgery of Shaoxing Second Hospital, a prefectural-level city tertiary hospital, analyze the influencing factors, and provide theories and data reference for clinical nursing practice and health education.

## Methods

### Subjects

In all, 161 patients discharged with tubes from the department of hepatobiliary surgery of Shaoxing Second Hospital from January 2017 to November 2019 were selected as the research subjects, and their discharge conditions were investigated. General information questionnaire, Chinese version of RHDS and Quality of Discharge Teaching Scale (QDTS) were filled in 4 h before discharge. There were 85 males and 76 females with an average age of 47.26 years old and an average hospital stay of 7.9 days. Inclusion criteria: a. Voluntary participation with informed consent; b. No cognitive dysfunction and communication barriers; c. No mental diseases; d. Age ≥ 18 years old. Exclusion criteria: a. Automatically discharged patients with unstable vital signs; b. With cognitive dysfunction. If the patients are illiterate and unable to complete the questionnaire independently, they will complete the questionnaire in the question–answer form with the cooperation of the researchers or the nurses in charge.

### General information questionnaire

General information included the patient's gender, age, BMI (body mass index), educational level, occupation, smoking history, drinking history, type of medical insurance, residence, family income per capita per month, caregiver after discharge, type of tubes, cathertering time before discharge, whether having performed abdominal surgeries and whether having benign or malignant diseases, etc.

### RHDS and QDTS

The RHDS used was the modified Chinese version one established by Weiss and Piacetine [15, 18] originally and improved by Taiwan scholar Lin [16] and Zhao [17] of the West China Hospital. There were 23 items in the scale, including 4 dimensions: Basic information (7 items), disease knowledge (8 items), extramural hospital coping capacity (3 items), and expected social support (4 items). Item 1 is a Y/N question not included in the total score. The rest of the questions are scored by 0–10 scores. 0 indicates that they are not ready to discharge at all, while 10 indicates that they completely get ready for discharge. Item 3 and 6 are reverse coded items and need reverse scoring. The total score is 220. The higher the total score, the higher the patient's readiness for discharge. The overall validity and Cronbach's α were 0.88 and 0.89, respectively. The Chinese version of the QDTS was also established by Weiss [18] originally, and assessed for validity by Binghua Wang et al. [19]. It was divided into three dimensions, including the information the patients required to know before discharge (6 items), the actual obtained information (6 items), discharge teaching skills and effects (12 items). Each item was scored by 0–10 scores, and the overall validity and Cronbach's α were 0.98 and 0.89, respectively.

### Statistical methods

Scale data entry and checking were completed by two persons, and the statistical analysis was conducted by SPSS19.0 software. The measurement data were expressed by‾χ ± s, and the mean values were compared by one-way ANOVA. The LSD-t test was used for pairwise comparison between groups, and Krusskal-Wallis H nonparametric test was used when the variance was not heterogeneous. Multiple linear regression analysis was performed, with the factors with statistical significance in the one-way ANOVA used as independent variables, while the score of the RHDS used as the dependent variable. Pearson correlation analysis was used to analyze the correlation between the readiness for hospital discharge and the quality of discharge teaching. The test standard is α = 0.05. When *p* < 0.05, the difference was considered statistically significant.

## Results

### The overall readiness of patients discharged with tubes from the department of hepatobiliary surgery

70.2% of the discharged patients from the department of hepatobiliary surgery subjectively indicated that they were ready for discharge. The total readiness score of the 161 patients ranged from 71 to 189, with an average of 142.40 ± 23.98, and the dimensions with scores from high to low were social support, disease-related knowledge, coping ability and basic information. The total score of the QDTS was 148.14 ± 17.74, and the dimensions with scores from high to low were the actual obtained information, required information, teaching skills and effect. The detailed scores of discharged patients with tubes in the department of hepatobiliary surgery are shown in Table 1.

**Table 1:** The total score of the RHDS and the QDTS and the score of each dimension of the patients discharged with tubes from the department of hepatobiliary surgery (n = 161)

Items	Full score	Item number	Actual score (‾χ ± s)	Average score of items (‾χ ± s)	Order
RHDS					
Social support	40	4	31.37 ± 2.30	7.84 ± 0.58	1
Disease-related knowledge	80	8	53.81 ± 15.88	6.72 ± 1.99	2
Coping ability	30	3	18.94 ± 6.40	6.31 ± 2.13	3
Basic information	70	7	38.29 ± 14.68	5.47 ± 2.10	4
Total	220	22	142.40 ± 23.98	6.45 ± 1.09	-
QDTS					
Actual obtained information	60	6	45.09 ± 2.54	7.52 ± 0.42	1
Required information	60	6	44.75 ± 2.60	7.46 ± 0.43	2
Teaching skills and effect	120	12	58.30 ± 17.17	4.86 ± 1.43	3
Total	240	24	148.14 ± 17.74	6.17 ± 0.74	-

### One-way ANOVA for the possible influencing factors of patients discharged with tubes

Factors, including age, smoking history, educational level, residence, type of tubes, cathetering time before discharge, abdominal surgeries, benign or malignant diseases, and the total time of hospitalization, all had a statistically significant effect on the readiness for hospital discharge of the patients discharged with tubes (*p* < 0.05), as shown in Table 2.

**Table 2:** Summary of influencing factors and one-way ANOVA results of the patients discharged with tubes (n = 161)

Items	Value	cases	Percentage	Score (‾χ ± s)	Statistics	P value
Gender					T = 1.534	0.125
Male	1	85	52.8%	145.47 ± 24.38		
Female	0	76	47.2%	139.66 ± 23.41		
Age					F = 4.085	0.019
< 25	1	14	8.7%	146.43 ± 22.90		
25 ≤ age ≤ 55	2	93	57.8%	146.13 ± 21.84		
> 55	3	54	33.5%	134.94 ± 26.33		
Smoking history					H = 5.453	0.020
No	0	81	50.3%	137.3 ± 26.73		
Yes	1	80	49.7%	147.57 ± 19.37		
Drinking history					T = -0.092	0.927
No	0	61	37.9%	142.18 ± 25.15		
Yes	1	100	62.1%	142.54 ± 23.36		
BMI					F = 2.520	0.084
< 18.5	1	6	3.7%	122.17 ± 25.86		
18.5 ≤ BMI ≤ 24	2	124	77.0%	142.50 ± 24.01		
> 24	3	31	19.3%	145.94 ± 22.28		
Marriage					T = -0.006	0.995
Married	1	133	82.6%	142.40 ± 24.80		
Others	0	28	17.4%	142.43 ± 20.02		
Educational level					F = 26.482	< 0.001
Primary school and below	1	27	16.8%	111.59 ± 17.86		
Junior	2	96	59.6%	148.52 ± 20.00		
Senior	3	24	14.9%	150.13 ± 20.62		
Vocational school and higher	4	14	8.7%	146.64 ± 19.66		
Occupation					H = 2.230	0.526
Permanent employment	1	60	37.3%	139.77 ± 27.71		
Part-time employment	2	10	6.2%	152.50 ± 12.67		
Retired	3	77	47.8%	143.30 ± 21.91		
Unemployed	4	14	8.7%	141.57 ± 23.61		
Provider payments					T = -0.082	0.935
With medical insurance	1	151	93.8%	142.44 ± 24.05		
Without medical insurance	0	10	6.2%	141.80 ± 24.12		
Main caregiver after discharge					F = 1.187	0.308
Self	1	17	10.6%	142.65 ± 21.36		
Immediate relatives	2	119	73.9%	140.97 ± 23.86		
Others	3	20	15.5%	149.08 ± 25.92		
Family income per capita per month					T = 0.963	0.337
< 3000	1	65	40.4%	144.62 ± 22.27		
≥ 3000	2	96	59.6%	140.91 ± 25.07		
Residence					H = 37.497	< 0.001
Rural	1	109	67.7%	134.46 ± 22.72		
Urban	2	52	32.3%	159.06 ± 17.10		
Type of tubes after discharge					F = 4.834	< 0.001
T tube	1	78	48.4%	133.59 ± 23.40		
PTCD tubes	2	26	16.1%	153.88 ± 16.98		
PTGD tubes	3	17	10.6%	152.53 ± 23.60		
Hepatophyma liver abscess drainage tubes	4	11	6.8%	147.55 ± 19.96		
Intraperitoneal drainage tubes	5	22	13.7%	147.77 ± 26.48		
Appendiceal abscess drainage tubes	6	7	4.3%	148.43 ± 20.74		
Status of disease					H = 12.771	< 0.001
Benign	0	111	68.9%	137.83 ± 24.99		
Malignant	1	50	31.1%	152.56 ± 17.97		
Cathetering time before discharge/Day					T = -5.353	< 0.001
< 5	1	121	75.2%	137.03 ± 23.14		
≥ 5	2	40	24.8%	158.65 ± 18.72		
Abdominal surgeries					H = 6.938	0.008
No	1	134	83.2%	140.10 ± 24.45		
Yes	0	27	16.8%	153.85 ± 17.79		
Total time of hospitalization/Day					H = 30.179	< 0.001
< 7	0	122	75.8%	136.50 ± 22.93		
≥ 7	1	39	24.8%	160.87 ± 16.85		

### Correlation analysis of the readiness for hospital discharge and the quality of discharge teaching of the patients discharged with tubes from the department of hepatobiliary surgery

**Fig. 1:**
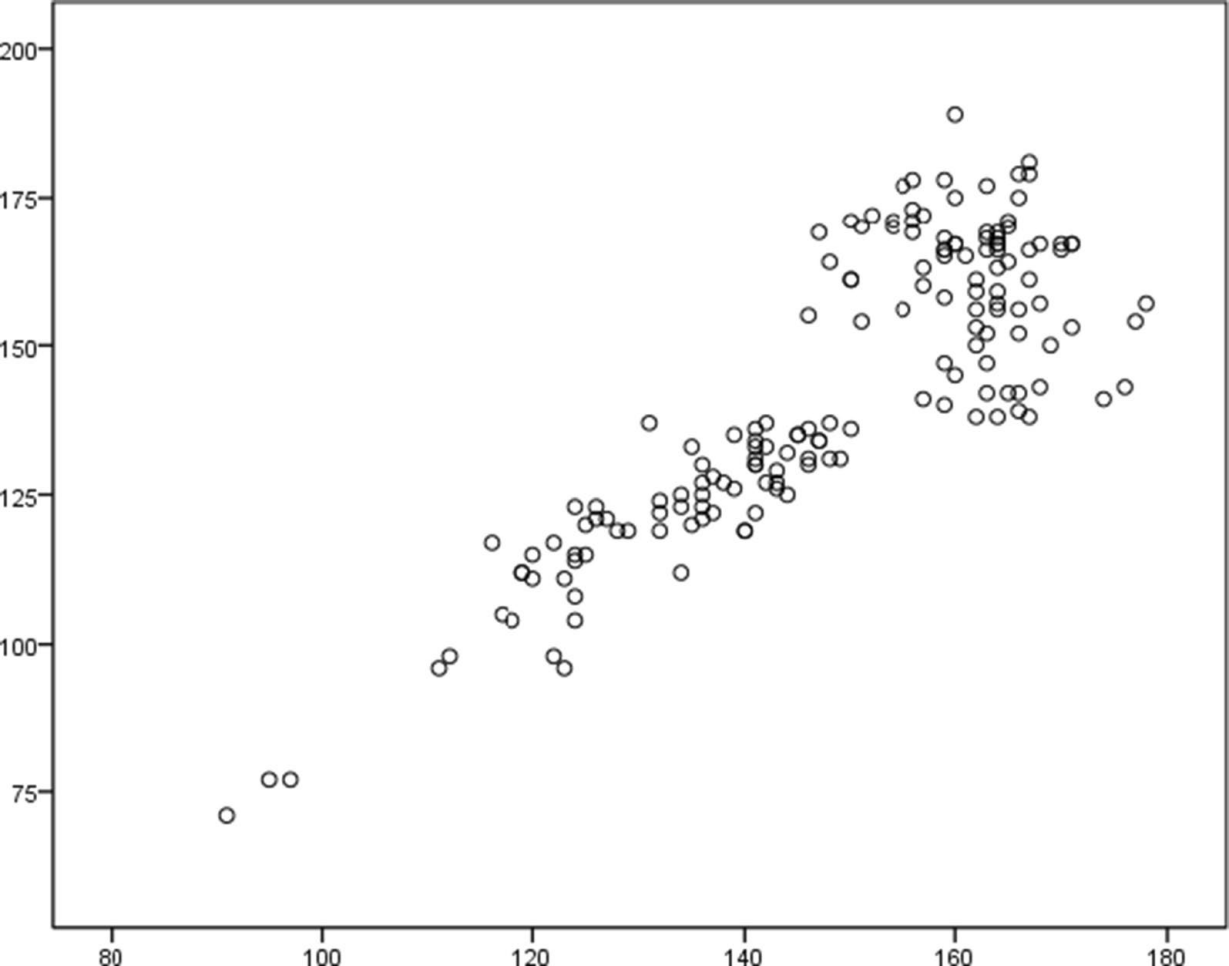
Correlation analysis of the readiness for hospital discharge and the quality of discharge teaching

The results showed that the total score of the RHDS was significantly correlated with the total score of the QDTS (R = 0.855, *p* < 0.001) (Fig. 1), as well as significantly correlated with the discharge teaching skills and effects (R = 0.889, *p* < 0.001). The analysis of subgroups showed that patient's basic condition, disease knowledge and coping capability were generally related to discharge teaching skills and effects, and the total score of the QDTS, as shown in Table 3.

**Table 3:** Correlation analysis of the readiness for hospital discharge and the quality of discharge teaching of patients discharged with tubes from the department of hepatobiliary surgery (n = 161)

Items		Required information	Actual obtained information	Discharge teaching skills and effects	Total score of the QDTS
Basic information	Pearson coefficient	− 0.073	− 0.009	0.562**	0.532**
	P	0.357	0.911	< 0.001	< 0.001
Disease-related knowledge	Pearson coefficient	− 0.050	0.063	0.658**	0.638**
	P	0.531	0.430	< 0.001	< 0.001
Coping capability	Pearson coefficient	0.020	0.018	0.459**	0.449**
	P	0.804	0.824	< 0.001	< 0.001
Social support	Pearson coefficient	0.001	− 0.023	− 0.135	− 0.134
	P	0.990	0.775	0.087	0.090
Total score of the RHDS	Pearson coefficient	− 0.072	0.039	0.889**	0.855**
	P	0.362	0.627	< 0.001	< 0.001

^**^The correlation at 0.05 level (two-sided test) is statistically significant

### The multiple linear regression analysis (Stepwise) of patients discharged with tubes

The total score of the RHDS was taken as the dependent variable, and the factors with statistical significance (*p* < 0.05) in one-way ANOVA were used as the independent variables. The stepwise multiple linear regression analysis was applied, and the assignment method is shown in Table 2, while the results of multiple linear regression are shown in Table 4. The results showed that the total score of the QDTS, the residence and the educational level were the main factors influencing the readiness for hospital discharge of the patients discharged with tubes (adjusted R-square = 0.768, F = 177.129, *p* < 0.000).

**Table 4:** Multiple linear regression analysis (Stepwise) (n = 161)

Independent variable	B	SE	β	t	P	95% CI
Constant	− 27.17	7.69		− 3.53	0.001	− 42.36 to − 11.97
Total score of the QDTS	1.01	0.06	0.75	17.12	0.000	0.90 to 1.13
Residence	10.41	2.10	0.20	4.97	0.000	6.27 to 14.55
Educational level	2.70	1.22	0.09	2.21	0.028	0.29 to 5.11

### One-way ANOVA analysis and multiple linear regression analysis (Stepwise) of patients discharged with T tube

In order to eliminate the potential biases caused by the diversity and heterogeneity of hepatobiliary diseases, we made further statistics of the subgroup patients which are discharged with T tube, since patients discharged with T tube accounted for most of the patients. The results showed that smoking history, educational level, residence, and the total time of hospitalization, had a statistically significant effect on the readiness for hospital discharge of the patients discharged with T tubes (p < 0.05), as shown in Table 5. The stepwise multiple linear regression analysis indicated that the educational level and the total time of hospitalization were the main factors influencing the readiness for hospital discharge of the patients discharged with T tube (adjusted R-square = 0.251, F = 13.917, p < 0.000), as shown in Table 6.

**Table 5:** Summary of influencing factors and one-way ANOVA results for discharged patients with T tube (n = 78)

Items	Value	cases	Percentage	Score (`c ± s)	Statistics	P value
Gender					t = 1.761	0.082
Male	1	43	55.1%	128.95 ± 22.19		
Female	0	35	44.9%	138.29 ± 24.57		
Age					F = 1.951	0.149
< 25	1	8	10.3%	134.75 ± 16.45		
25 ≤ age ≤ 55	2	41	52.6%	137.56 ± 21.81		
> 55	3	29	37.2%	126.45 ± 26.61		
Smoking history					t = -2.565	0.012
No	0	41	52.6%	126.85 ± 25.98		
Yes	1	37	47.4%	140.11 ± 18.60		
Drinking history					t = 1.043	0.300
No	0	35	44.9%	136.23 ± 24.84		
Yes	1	43	55.1%	130.63 ± 22.52		
BMI					F = 2.877	0.063
< 18.5	1	4	5.1%	106.75 ± 12.97		
18.5 ≤ BMI ≤ 24	2	61	78.2%	133.98 ± 24.47		
> 24	3	13	16.7%	137.31 ± 16.82		
Marriage					t = 0.269	0.790
Married	1	63	80.8%	132.87 ± 25.18		
Others	0	15	19.2%	134.27 ± 15.90		
Educational level					H = 20.25	0.000
Primary school and below	1	15	19.2%	107.00 ± 20.57		
Junior	2	44	56.4%	140.64 ± 21.55		
Senior	3	11	14.1%	136.00 ± 16.66		
Vocational school and higher	4	8	10.3%	137.00 ± 13.50		
Occupation					F = 1.684	0.178
Permanent employment	1	32	41.0%	130.44 ± 28.10		
Part-time employment	2	5	6.4%	154.80 ± 12.99		
Retired	3	35	44.9%	133.40 ± 20.14		
Unemployed	4	6	7.7%	128.00 ± 15.414		
Provider payments					t = -0.881	0.381
With medical insurance	1	74	94.9%	133.69 ± 23.99		
Without medical insurance	0	4	5.1%	123.00 ± 11.76		
Main caregiver after discharge					F = 0.542	0.584
Self	1	7	9.0%	140.51 ± 18.87		
Immediate relatives	2	59	75.6%	131.68 ± 22.79		
Others	3	12	15.4%	136.00 ± 30.15		
Family income per capita per month					t = 1.036	0.303
< 3000	1	31	39.7%	136.55 ± 19.57		
≥ 3000	2	47	60.3%	130.89 ± 25.87		
Residence					t = 3.381	0.001
Rural	1	37	47.4%	131.56 ± 29.33		
Urban	2	41	52.6%	151.87 ± 23.65		
Status of disease					t = -0.813	0.419
Benign	0	74	94.9%	132.64 ± 24.04		
Malignant	1	4	5.1%	142.50 ± 10.472		
Cathetering time before discharge/Day						
< 5	1	0	0.0%	/		
≥ 5	2	78	100.0%			
Abdominal surgeries						
No	1	0	0.0%	/		
Yes	0	78	100.0%			
Hospitalization/Day					t = -4.158	0.000
< 7	0	71	91.0%	129.97 ± 22.17		
≥ 7	1	7	9.0%	165.29 ± 9.25

**Table 6:** Multiple linear regression analysis (Stepwise) for discharged patients with T tube (n = 78)

Independent variable	B	SE	β	T	P	95% CI
Constant	112.67	6.32		17.82	0.000	100.08–125.27
Educational level	8.08	2.73	0.38	2.96	0.004	2.65–13.52
Hospitalization/Day	34.14	8.10	0.42	4.22	0.000	18.01–50.28

## Discussion

### The score of the RHDS of the discharged patients with tubes in the department of hepatobiliary surgery is in the middle and lower level

In this study, the score for discharge readiness was 142.40 ± 23.98 and the average score for items was 6.45 ± 1.09. The average score for items ranged from 3.23 to 8.59, with 102 people (63.35%) having an average score for items greater than or equal to 6. Compared with the reported readiness of patients with other clinical characteristics [20–22], the readiness of patients in this study was in the lower level (see Table 7), which is comparatively in line with the current situation of the readiness in grassroots hospitals. It provides certain data support for the grassroots hospitals to further learn from the hospitals of higher levels, strengthen admission education and discharge teaching. In this study, 78 patients (48.4%) were discharged with a T-tube after choledocholithotomy, and they were the main discharged patients with tubes in the department of hepatobiliary surgery in our hospital. The score of the readiness for these patients was 133.59 ± 23.40, which was significantly lower than that of other groups, and the difference was statistically significant. The reason might be presented in two aspects. On the one hand, compared with patients having had major operations for liver cancer and other diseases, the average hospitalization time of the patients in our study was significantly shortened, and the time from ECG monitoring to secondary nursing after operation was relatively short, which led to a decreased time of effective education in hospital, ultimately resulting in the phenomenon that discharge teaching was relatively insufficient. On the other hand, for most patients with benign diseases who needed to be readmitted for T-tube removal operation in this study, as the arrangement of hospitalization time and work procedure of our department is relatively perfect and mature, they had good compliance and thought that they only need to follow the arrangement of medical staff and there was no need to go much into disease-related professional knowledge. Such being the case, there was a certain degree of dependence and carelessness. While for patients discharged from hospital with PTCD tubes and PTGD tubes, most of them suffered from malignant diseases, or were hospitalized for many times with a long course of disease and repeatedly admitted to hospital for palliative treatment or supportive treatment. In this vein, patients and their families had a clear understanding of the disease itself and prognosis, and their readiness for hospital discharge was in a relatively high level.

**Table 7:** Horizontal comparison of the readiness for hospital discharge of patients with different clinical characteristics

Discharge readiness study	Number of patients	Items	Total score (‾χ ± s)	Average score for items (‾χ ± s)
1. Colorectal cancer patients underwent ERAS [20]^a^	130	22	149.86 ± 33.65	6.81 ± 1.53
2. Cataract patients treated with day surgery [21]^a^	194	22	175.51 ± 26.75	7.98 ± 1.22
3. Patients undergoing laryngectomy [22]^a^	202	22	–	7.76 ± 1.76
4. Patients with spinal cord injury [23]^a^	50	21	150.78 ± 27.06	–
This study	161	22	142.40 ± 23.98	6.45 ± 1.09

^a^Studies related to discharge readiness

### Influencing factors related to the readiness for hospital discharge of the patients discharged with tubes from the department of hepatobiliary surgery

According to the standardized regression coefficient, the total score of the QDTS, residence, and educational level were the three factors with a relatively large effect from high to low on the readiness for the patients discharged with tubes from the department of hepatobiliary surgery.

#### The total score of the QDTS is an important factor affecting the readiness of the patients discharged with tubes from the department of hepatobiliary surgery

According to the results of Pearson correlation analysis, the total score of the RHDS of the patients discharged with tubes from the department of hepatobiliary surgery of our hospital was significantly correlated with the total score of the QDTS, and was also significantly correlated with the discharge teaching skills and effects. In the meantime, according to the multiple linear regression, the standardized regression coefficient of the total score of the QDTS was 0.75. Both of the above findings suggested that we should do a good job of discharge teaching and it is conducive to improving the score of RHDS. Additionally, although the overall readiness for the patients discharged from the department of hepatobiliary surgery of our hospital was in a middle or lower level compared with that of the patients with other clinical characteristics, the regular pattern revealed by this study is objective truth consistent with previous reports. A variety of forms of discharge teaching for the patients with tubes before discharge have been carried out in the department of hepatobiliary surgery in our hospital: (a) Formulate standardized health education manual of nursing work in the department according to the disease type, and specify the specific education object, content, form and time; (b) Design of preoperative education for different drainage tubes and postoperative guidance for discharge, strengthen the propaganda and education for the patients who need to discharge with tubes, establish a doctor-patient evaluation system and record the actual time of each patient receiving the education, so as to facilitate the follow-up retrospective analysis and continuous quality improvement; (c) Standardize the standard fixation methods of various drainage tubes, and evaluate the slippage risk of all kinds of tubes in discharged patients with tubes according to the previously reported [24]. According to the slippage risk, different degrees and frequencies of psychological intervention before discharge and follow-up visit after discharge are implemented; (d) Establish a department level doctor-patient interaction WeChat platform to facilitate timely follow-up visit and feedback of discharged patients; meanwhile, carry out continuous nursing services in the community hospitals around the patients, so as to eliminate the psychological concerns of patients when they are discharged with tubes; (e) For patients who need to be re-hospitalized for tube removal, the time of re-admission should be informed before discharge, and the re-admission appointment should be completed to facilitate the processes between discharge and re-admission. Discharge teaching is an activity jointly participated by doctors and patients. Its effect is based on the health guidance and education in the whole process of hospitalization. Only by doing every detail well can patients leave the hospital calmly, thus improving the level of the readiness for hospital discharge and making patients calmly adapt to the life after discharge.

#### Residence and educational level also affect the readiness for hospital discharge of the patients discharged with tubes from the department of hepatobiliary surgery to a certain extent

This study showed that the readiness for the urban residents was significantly higher than that of the rural residents. According to the one-way ANOVA, age and educational level had a significant influence on the readiness of the discharged patients with tubes. Through subgroup analysis, there was no significant difference in age (chi-square value = 0.454, *p* > 0.05) and educational level (chi-square value = 5.449, *p* > 0.05) between the urban residents and the rural residents. Therefore, age and educational level cannot be used to explain the difference in the readiness between the urban residents and the rural residents. In multiple linear regression, both residence and educational level were included in the statistical model, and the confounding factors between them were eliminated. It is concluded that residence is an independent factor that can affect the readiness of the patients discharged with tubes from the department of hepatobiliary surgery. As far as the department of hepatobiliary surgery of our hospital is concerned, our hospital is located in the city, the distance between urban residents and the hospital is relatively close, and it is relatively convenient to go for medical treatment, subsequent visit and tube maintenance, so there are less concerns and psychological burden when leaving the hospital. That is why the readiness for hospital discharge of the urban residents is relatively high. To some extent, educational level determines the cognitive ability and psychological endurance. The intra group pairwise comparison showed that the discharge readiness of patients with primary school educational level and below was significantly lower than that of the patients with junior, senior and vocational school and higher educational levels, and the difference was statistically significant (*p* < 0.001 for pairwise comparison). Some patients with primary school educational level and below even needed the help and the guidance of medical staff when completing the RHDS. While the patients with a high educational level usually could complete the questionnaire independently and skillfully. After discharge teaching, they had a high level of compliance and self-discipline, and the readiness for discharge was therefore relatively high. In the future, for the discharge teaching of patients with a low educational level, on the one hand, more straightaway language and form should be considered, as well as more vivid metaphor, or even dialect; on the other hand, we can provide discharge teaching for the family members or caregivers of such patients with a higher educational level, so as to achieve the purpose of indirect guidance and improve the discharge readiness of patients with tubes.

## Conclusion

Study on the readiness for hospital discharge of the patients discharged from the department of hepatobiliary surgery with tubes under the fast track surgery is of great significance to reduce the occurrence of accidental extubation, tube-related infection, tube blockage and other adverse events after discharge. The results of this study show that: the total score of the quality of discharge teaching, residence and educational level are the important factors affecting the readiness of the patients discharged with tubes in the department of hepatobiliary surgery. Improving the quality of discharge teaching can significantly improve the level of the readiness of patients. Besides, the distance from the hospital and low educational level have a negative impact on the discharge readiness. In the future, when carrying out health education and discharge teaching, for patients far away from the hospital and with a low educational level, we should pay more attention to the actual effect, and timely adjust the form of education and teaching, so as to improve the level of the discharge readiness of relatively vulnerable patients, reduce the incidence of adverse events after discharge with tubes, and ensure the health and safety of patients.

## Data Availability

The data and materials in this current study are available from the corresponding author on reasonable request.

## Abbreviations

RHDS: Readiness for Hospital Discharge Scale
QDTS: Quality of Discharge Teaching Scale
BMI: Body mass index
PTCD: Percutaneous transhepatic cholangial drainage
PTGD: Percutaneous transhepatic gallbladder drainage
